# Four-And-A-Half LIM-Domain Protein 2 (FHL2) Deficiency Aggravates Cholestatic Liver Injury

**DOI:** 10.3390/cells9010248

**Published:** 2020-01-19

**Authors:** Judith Sommer, Christoph Dorn, Erwin Gäbele, Frauke Bataille, Kim Freese, Tatjana Seitz, Wolfgang E. Thasler, Reinhard Büttner, Ralf Weiskirchen, Anja Bosserhoff, Claus Hellerbrand

**Affiliations:** 1 Institute of Biochemistry, Emil-Fischer-Zentrum, Friedrich-Alexander University Erlangen-Nürnberg, Fahrstr. 17, D-91054 Erlangen, Germany; judith.sommer@fau.de (J.S.); kim.freese@fau.de (K.F.); tatjana.seitz@fau.de (T.S.); anja.bosserhoff@fau.de (A.B.); 2 Institute of Pharmacy, University Regensburg, D-93053 Regensburg, Germany; christoph.dorn@chemie.uni-regensburg.de; 3 Department of Internal Medicine I, University Hospital Regensburg, D-93053 Regensburg, Germany; erwin.gaebele@ukr.de; 4 Institute of Pathology, University Regensburg, D-93049 Regensburg, Germany; bataillef@t-online.de; 5 Hepacult GmbH, D-82152 Planegg/Martinsried, Germany; Wolfgang.Thasler@hepacult.de; 6 Institute of Pathology, University Hospital Cologne, D-50937 Cologne, Germany; reinhard.buettner@uk-koeln.de; 7 Institute of Molecular Pathobiochemistry, Experimental Gene Therapy and Clinical Chemistry, RWTH University Hospital Aachen, D-52074 Aachen, Germany; rweiskirchen@ukaachen.de; 8 Comprehensive Cancer Center (CCC) Erlangen-EMN, D-91054 Erlangen, Germany

**Keywords:** four-and-a-half LIM-domain protein 2, FHL2, cholestatic liver injury, bile acids

## Abstract

Cholestasis occurs in different clinical circumstances and leads to severe hepatic disorders. The four-and-a-half LIM-domain protein 2 (FHL2) is a scaffolding protein that modulates multiple signal transduction pathways in a tissue- and cell context-specific manner. In this study, we aimed to gain insight into the function of FHL2 in cholestatic liver injury. FHL2 expression was significantly increased in the bile duct ligation (BDL) model in mice. In *Fhl2*-deficient (*Fhl2*-ko) mice, BDL caused a more severe portal and parenchymal inflammation, extended portal fibrosis, higher serum transaminase levels, and higher pro-inflammatory and pro-fibrogenic gene expression compared to wild type (wt) mice. FHL2 depletion in HepG2 cells with siRNA resulted in a higher expression of the bile acid transporter Na^+^-taurocholate cotransporting polypeptide (*NTCP*) gene. Furthermore, FHL2-depleted HepG2 cells showed higher expression of markers for oxidative stress, lower B-cell lymphoma 2 (Bcl2) expression, and higher Bcl2-associated X protein (*BAX*) expression after stimulation with deoxycholic acid (DCA). In hepatic stellate cells (HSCs), FHL2 depletion caused an increased expression of TGF-β and several pro-fibrogenic matrix metalloproteinases. In summary, our study shows that deficiency in FHL2 aggravates cholestatic liver injury and suggests FHL2-mediated effects on bile acid metabolisms and HSCs as potential mechanisms for pronounced hepatocellular injury and fibrosis.

## 1. Introduction

Fibrosis is a highly conserved protective response to tissue injury. It is an essential biological mechanism for the maintenance of structural and functional tissue integrity. Additionally, hepatic fibrosis can be considered as a wound-healing response to liver injury. It reflects a balance between liver repair and scar formation. Pathological fibrosis corresponds to impaired wound healing [1]. The liver usually regenerates after an acute liver injury, but, if the injury persists, the liver reacts with progressive inflammatory response and fibrogenesis. The progressive and uncontrolled accumulation of extracellular matrix (ECM) proteins eventually leads to liver cirrhosis and hepatic failure. Furthermore, liver cirrhosis is a major risk factor for the development of hepatocellular cancer (HCC) [2,3]. Therefore, hepatic fibrosis is the most important pathophysiological factor that determines the morbidity and mortality of patients with chronic liver disease [4,5]. Unfortunately, no effective anti-fibrogenic therapy has been established thus far. Accordingly, there is a high medical need to improve our understanding of pathogenic mechanisms underlying hepatic fibrogenesis in order to identify new targets and to develop therapeutic strategies to inhibit hepatic fibrosis in patients with chronic liver disease.

The four-and-a-half LIM-domain protein 2 (FHL2), also known as SLIM3 or DRAL, is the second member of the four-and-a-half LIM-domain protein family [6]. LIM proteins mediate protein–protein interactions, and FHL2 has also been described to interact with more than 50 different proteins that are involved in different signaling pathways [7]. Consequently, FHL2 has been shown to play important roles in various biological mechanisms, including cell proliferation, apoptosis, and transformation [7,8]. FHL2 is ubiquitously expressed, but it exerts tissue- and cell context-specific functions that are often opposing. For example, FHL2 inhibits the migration of dendritic cells, but it induces the migration of fibroblasts [9]. FHL2 has also been shown to affect different steps of wound healing [9]. The deletion of the *Fhl2* gene in mice has been found to result in impaired wound healing in the skin, the intestine or ischemic muscle tissues [10,11,12,13]. So far, little information exists regarding the role of FHL2 in the liver and chronic liver diseases.

Cholestatic liver injury is a pathophysiological situation that is relevant for a variety of diseases such primary biliary cholangitis, primary biliary sclerosis, and drug-induced hepatotoxicity. Cholestasis leads to hepatocellular injury and subsequent inflammation and fibrosis. Still, the molecular mechanisms and interplay between different pathological effects and cell types that lead to disease progression are only incompletely understood.

The aim of this study was to analyze the role of FHL2 in cholestatic liver injury with a focus on hepatocellular damage and fibrosis.

## 2. Materials and Methods

### 2.1. Cells and Cell Culture

The hepatoma cell line HepG2 (ATCC HB-8065) and the human hepatic stellate cell line LX-2 were cultured as described in [14]. The isolation and culture of primary human hepatic stellate cells (HSCs) was performed as described in [15]. Human liver tissue for cell isolation was obtained from the charitable, state-controlled Human Tissue and Cell Research (HTCR) foundation [16] with informed patient’s consent.

### 2.2. FHL2 Depletion with siRNA-Pools

Transfection with FHL2 siRNA-pools was performed as described in [17] by using the Lipofectamine RNAimax transfection reagent (Life Technologies, Darmstadt, Germany) and siRNA-pools against human FHL2 mRNA (functionally verified by siTOOLs Biotech GmbH, Planegg, Germany). Si-pools are complex pools of defined siRNAs that are directed against the target gene, leading to a robust knockdown, while off-target effects are believed to be significantly reduced [18]. At 72 h after transfection, cells were further analyzed. For stimulation experiments, HepG2 cells were treated with deoxycholic acid (DCA) (Sigma-Aldrich, Steinheim, Germany) for 24 h at indicated concentrations. Cytotoxic effects were monitored by the analysis of lactate dehydrogenase (LDH) release into the supernatant by using the Pierce LDH cytotoxicity assay kit (Thermo Fisher Scientific, Waltham, MA, USA).

### 2.3. Animals and Bile Duct Ligation

Male *Fhl2*-ko mice [13,19] and wild type (wt) littermates aged 10–12 weeks were kept under controlled standard conditions. The mice were fed with a standard laboratory diet and had access to water ad libitum. Bile duct ligation (BDL) and sham operations (control animals) were performed as described (*n* = 5 animals/group) [20]. The animal studies were approved by the Committee for Animal Health and Care of the local government (54-2531.1-28/05) and conformed to international guidelines on the ethical use of animals. After 2 weeks, animals were sacrificed, and blood samples were collected. Liver tissue samples were either fixed in 5% formalin or snap-frozen in liquid nitrogen and stored at −80 °C until subsequent analyses.

### 2.4. Quantitative Real-Time-PCR Analysis

RNA isolation from liver tissues and reverse transcription were performed as described in [21]. Quantitative real-time PCR was performed by applying LightCycler technology (Roche Diagnostics, Mannheim, Germany) while using specific sets of primers, as listed in Table 1. For the detection of the human *NTCP*, *p47phox* and *TGF-β* genes, QuantiTect Primer Assays (Qiagen, Hilden, Germany) were used. For normalization, the amplification of cDNA derived from *18S* rRNA was used.

### 2.5. Protein Analysis

Protein extraction from liver tissues, protein extraction from cells, and analysis by Western blotting were performed as described in [17] by applying mouse monoclonal anti-FHL2 (HycultBiotech, Uden, The Netherlands; HM2136, 1:300), rabbit monoclonal anti-alpha-smooth muscle actin (α-SMA) (Abcam, Cambridge, United Kingdom; ab32575, 1:1000), rabbit monoclonal anti-B-cell lymphoma 2 (BCL2) (Epitomics, Burlingame, CA, USA; #1017, 1:1000), rabbit polyclonal anti-metalloproteinase 13 (anti-MMP13) (Abcam, Cambridge, United Kingdom; ab39012, 1:1000), rabbit polyclonal anti-MMP14 (Chemicon, Burlington, MA, USA; AB815, 1:1000) and mouse monoclonal anti-actin (ACTB; Merck Millipore, Billerica, MA, USA; MAB1501, 1:10,000) antibodies.

### 2.6. (Immono)Histological Analysis

For hematoxylin and eosin (HE) staining, Sirius Red/Fast Green staining and immunohistological analysis, formalin-fixed and paraffin-embedded tissue blocks were sectioned at a standard thickness of 5 µm, deparaffinized with xylene, and stained as described previously [22] by applying the following antibodies: rabbit monoclonal anti-α-SMA (Abcam, Cambridge, United Kingdom; ab32575, 1:300) and rabbit polyclonal anti-CD3 (Sigma-Aldrich, St. Louis, MO, USA; C7930, 1:1000). Sirius Red/Fast Green staining was performed as described previously [23]. Microscopical images were taken with an Olympus^TM^ CKX41 microscope with the ALTRA 20 Soft Imaging System^TM^ and Cell^A^ software version 2.6 (Olympus Soft Imaging Solutions GmbH, Münster, Germany). IrfanView^TM^ software version 4.36 (Irfan Skiljan, Jajce, Bosnia) was used in order to process images.

### 2.7. Statistical Analysis

Values are presented as mean ± SEM. Student’s unpaired t-test or, when appropriate, a two-way ANOVA test with the Sidak correction were used for comparison between groups, and a *p*-value < 0.05 was considered statistically significant. All analyses were performed at least in triplicates. Calculations were performed by applying the statistical computer package GraphPad Prism version 6.01 for Windows (GraphPad Software, San Diego, CA, USA).

## 3. Results

### 3.1. Fhl2 Deficiency Aggravates Hepatocellular Injury and Inflammation in Cholestatic Liver Injury

**Figure 1:**
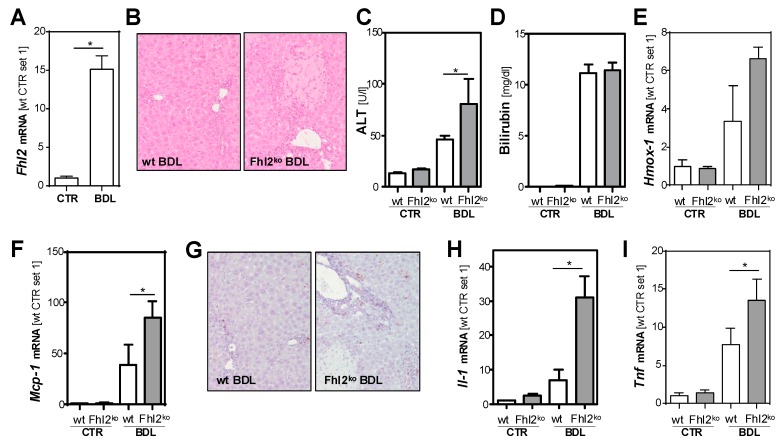
*Fhl2* expression and effect of *Fhl2* deficiency on hepatocellular injury and inflammation in the mouse model of bile duct ligation (BDL). *Fhl2*-deficient (*Fhl2*-ko) and wild type (wt) mice were either bile duct ligated (BDL) or sham-operated (CTR). (**A**) *Fhl2* mRNA levels in wt BDL and CTR mice analyzed by qRT-PCR. (**B**) Representative hematoxylin and eosin stainings of liver tissue samples (20× magnification). (**C**) ALT (alanine aminotransferase) and (**D**) bilirubin serum levels. (**E**) *Hmox-1* and (**F**) *Mcp-1* mRNA expression levels in liver tissue analyzed by qRT-PCR. (**G**) Immunohistochemical CD3 staining of liver tissue samples (20× magnification). (**H**) *Il-1* and (**I**) *Tnf* mRNA expression levels in liver tissue analyzed by qRT-PCR. (*: *p* < 0.05).

The experimental obstruction of the extrahepatic biliary system initiated a pathological cascade of events that led to cholestasis-induced hepatocellular injury and inflammation, which resulted in a strong fibrogenic reaction. Initially, we analyzed hepatic *Fhl2* expression two weeks after the surgical ligation of the common bile duct in mice and observed a markedly increased upregulation as compared to sham-operated control mice (Figure 1A).

Subsequently, we applied this model of bile duct ligation (BDL) to male *Fhl2*-deficient (*Fhl2*-ko) and wt littermates. The sham-operated *Fhl2*-ko and wt mice served as controls. In response to BDL, the wt mice showed some inflammatory infiltration and few distinct necrotic areas (Figure 1B). In contrast, large necrotic areas and pronounced parenchymal inflammation appeared in *Fhl2*-ko mice with BDL in the histological analysis (Figure 1B). Fitting this, the BDL-induced increase of alanine aminotransferase (ALT) levels was significantly higher in the *Fhl2*-deficient mice compared to the wt mice (Figure 1C). In contrast, serum bilirubin levels were markedly increased in both the wt and *Fhl2*-deficient mice and to a similar extent (Figure 1D).

Furthermore, the hepatic expression of heme oxygenase 1 (*Hmox-1*), a marker of oxidative stress, was markedly increased in response to BDL (Figure 1E). The BDL-induced *Hmox-1* expression levels tended to be higher in the *Fhl2*-deficient mice compared to the wt mice, further indicating that there was more pronounced hepatocellular injury in the *Fhl2*-deficient mice. The chemokine monocyte chemotactic protein 1 (*Mcp-1*) is produced at sites with oxidative stress and attracts inflammatory cells [24]. Fitting this, the BDL-induced expression levels of *Mcp-1* were significantly higher in the *Fhl2*-deficient mice compared to the wt mice (Figure 1F), and CD3-immunohistochemical staining revealed a strong lymphocytic infiltration in the *Fhl2*-deficient BDL mice but only a few CD3-positive cells in the liver tissue of the wt-mice with BDL (Figure 1G). Moreover, the BDL-induced expression levels of the pro-inflammatory cytokines *Il-1* and *Tnf* were significantly higher in the *Fhl2*-deficient mice compared to the wt mice (Figure 1H,I).

In summary, *Fhl2* deficiency in mice promoted hepatocellular injury and inflammation in the BDL model of cholestatic liver injury.

### 3.2. Fhl2 Deficiency Aggravates Hepatic Fibrosis in the Mouse Model of Bile Duct Ligation

**Figure 2:**
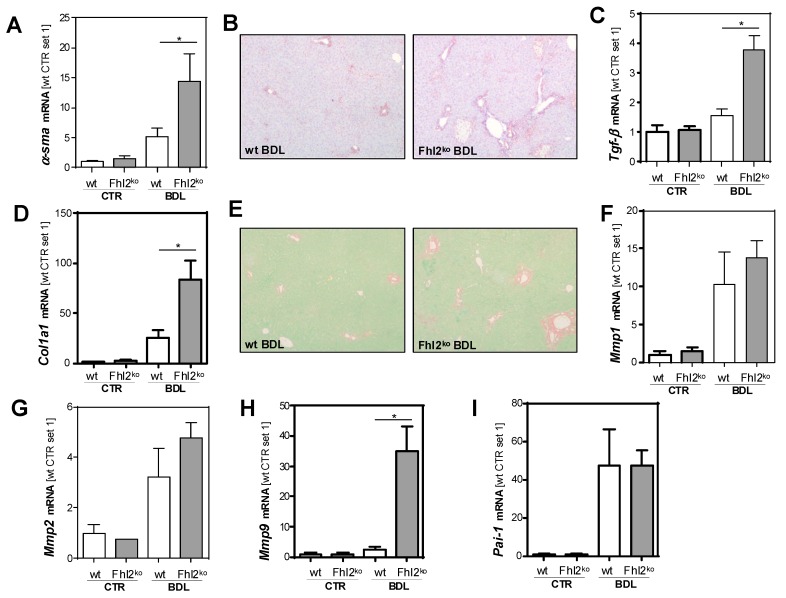
Effect of *Fhl2* deficiency on hepatic fibrosis in the mouse model of bile duct ligation (BDL). *Fhl2*-deficient (*Fhl2*-ko) and wild type (wt) mice were either subjected to BDL or were sham-operated (CTR). (**A**) Hepatic *α-sma* mRNA expression levels. (**B**) Immunohistochemical α-sma staining of liver tissue sections (20× magnification). Hepatic (**C**) *Tgf-β* and (**D**) *Col1a1* mRNA expression levels. **(E)** Sirius Red/Fast Green staining of liver tissue sections (20× magnification). Hepatic (**F**) *Mmp1*, (**G**) *Mmp2*, (**H**) *Mmp9*, and (**I**) *Pai-1* mRNA expression levels in liver tissue analyzed by qRT-PCR. (*: *p* < 0.05).

Activated hepatic stellate cells are a major cellular source of MCP-1 in injured livers [15]. In line with this, the expression of α-smooth muscle actin (*α-sma*), a marker for HSC activation, was upregulated in response to BDL, with significantly higher expression levels in the *Fhl2*-deficient mice than the wt mice (Figure 2A). An immunohistochemical analysis confirmed strong α-sma staining in the liver tissue of the *Fhl2*-ko mice with BDL, while the few α-sma positive cells in the wt-mice with BDL were mainly located around the periportal fields (Figure 2B). Furthermore, the expression of transforming growth factor-β (*Tgf-β*), the most prominent pro-fibrogenic cytokine in liver fibrosis, was significantly higher in the livers of *Fhl2*-deficient compared to the wt mice that were exposed to BDL (Figure 2C). Fitting this, the *Fhl2*-ko mice with BDL showed significantly higher expression levels of Collagen type 1 α I (*Col1a1*) as compared to the wt mice with BDL (Figure 2D). Sirius Red staining confirmed enhanced extracellular matrix deposition in the *Fhl2*-ko-BDL mice compared to the wt-BDL mice (Figure 2E). Additionally, the expression levels of the matrix metalloproteinases (*Mmp*) 1 and 2 was markedly increased in response to BDL, with a high variation in expression levels in the *Fhl2*-deficient and wt animals (Figure 2F,G). In contrast, the expression of *Mmp9* was only slightly higher in the wt mice with BDL compared to the sham-operated littermates (Figure 2H). However, in the *Fhl2*-ko mice, BDL caused a marked increase of hepatic *Mmp9* expression. On the contrary, the expression of plasminogen activator inhibitor 1 (*Pai-1*) was manifestly increased in both the *Fhl2*-ko and wt mice with BDL (Figure 2I).

Still and, in summary, these data clearly indicate that mice with *Fhl2* deficiency were more prone to hepatic fibrosis in the BDL model.

### 3.3. FHL2 Depletion Promotes Bile Acid-Induced Hepatocellular Injury In Vitro

**Figure 3:**
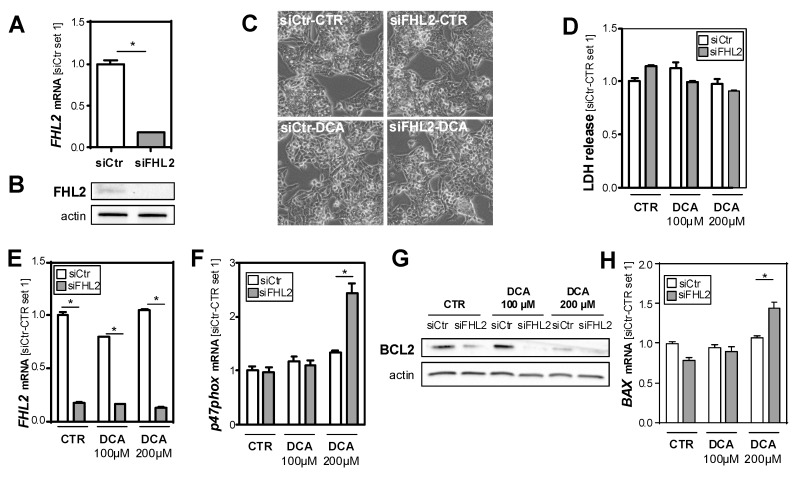
Effect of FHL2 depletion on bile acid-induced hepatocellular injury in vitro. (**A**,**B**) *FHL2* mRNA and protein expression in HepG2 cells transfected with si-pools against FHL2 (siFHL2) and si-control-pools (siCtr). (**C**) Representative microscopical images 72 h after transfection (10X magnification). (**D**) Quantification of lactate dehydrogenase (LDH) release into the supernatant. (**E**) *FHL2*, (**F**) *p47phox*, and (**H**) *BAX* mRNA, and (**G**) B-cell lymphoma 2 (BCL2) protein expression after treatment with deoxycholic acid (DCA) for 24 h and control cells (CTR). (*: *p* < 0.05).

To gain further insight into the mechanism by which FHL2 affects hepatocellular injury in the BDL-induced model of chronic cholestasis, we wanted to analyze whether FHL2 affected the cellular response of hepatocytes to bile acids in vitro. To achieve this, we used the well-established human hepatoma cell line HepG2 and si-pool technology for specific FHL2 suppression (Figure 3A,B). Subsequently, cells were treated with two different concentrations of deoxycholic acid (DCA) for 24 h. DCA exposition did not affect FHL2 expression levels (Figure 3E). Furthermore, we did not observe cytotoxic effects under these experimental conditions (Figure 3C,D). However and interestingly, the highest dose of DCA induced a marked induction of neutrophil cytosolic factor 1 (p47phox) in the FHL2-depleted cells, while the expression levels did not significantly change in the control transfected cells (Figure 3F). p47phox is a regulatory subunit of nicotinamide adenine dinucleotide phosphate oxidases (NOXs) and a well-known marker of oxidative stress in hepatocytes [25]. Furthermore, it has been shown to be critically involved in bile salt-induced apoptosis [26]. Fitting this, western blot analysis revealed lower expression of B-cell lymphoma 2 (*BCL2*) in FHL2-depleted cells that further decreased in response to DCA stimulation (Figure 3G). In contrast, the mRNA expression of Bcl2-associated X protein (*BAX*) increased in DCA-treated cells with FHL2 suppression, while the expression levels of this key factor of intrinsic apoptosis did not change in control cells. (Figure 3H). These data suggest that FHL2 protected HepG2 cells from bile acid-induced oxidative stress and apoptosis.

### 3.4. FHL2 Depletion Affects Expression of Key Enzymes of Bile Acid Metabolism

**Figure 4:**
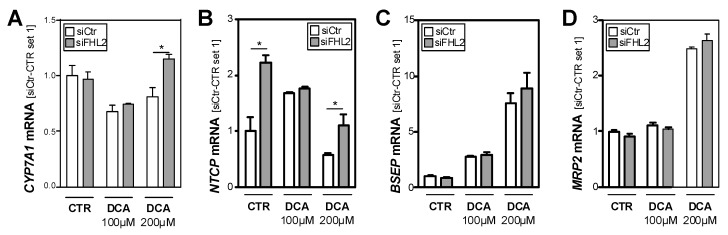
Effect of FHL2 depletion on bile acid metabolism. HepG2 cells were transfected with si-pools against FHL2 (siFHL2) and si-control-pools (siCtr). Analysis of (**A**) *CYP7A1*, (**B**) *NTCP*, (**C**) *BSEP*, and (**D**) *MRP2* mRNA expression after treatment with deoxycholic acid (DCA) for 24 h and control cells (CTR). (*: *p* < 0.05).

Cytochrome P450 7A1 (CYP7A1) is the key enzyme of bile acid synthesis. Its expression levels were slightly reduced in the DCA-treated control cells, while the FHL2-depleted cells showed increased expression levels in response to the highest DCA dose (Figure 4A). Interestingly, Na^+^-taurocholate cotransporting polypeptide (*NTCP*), the major transporter for bile acid uptake, was already, under basal conditions, significantly higher expressed in FHL2-depleted compared to control cells (Figure 4B). In response to DCA stimulation, *NTCP* expression levels declined but remained higher in the FHL2-depleted cells (Figure 4B).

In contrast, the expression levels of bile salt export pump (*BSEP*) and multidrug resistance-associated protein 2 (*MRP2*) did not differ between the FHL2-depleted and control cells (Figure 4C,D).

Moreover, the exposition to DCA caused a marked induction of the expression of these major bile salt export transporters, but this induction was similar in the FHL2-depleted and control cells. Together, these data indicate that the elevated synthesis and uptake of bile acids in FHL2-depleted cells may be a potential cause for enhanced hepatocellular injury in cholestasis.

### 3.5. FHL2 Depletion Promotes Pro-Fibrogenic Gene Expression in Hepatic Stellate Cells

**Figure 5:**
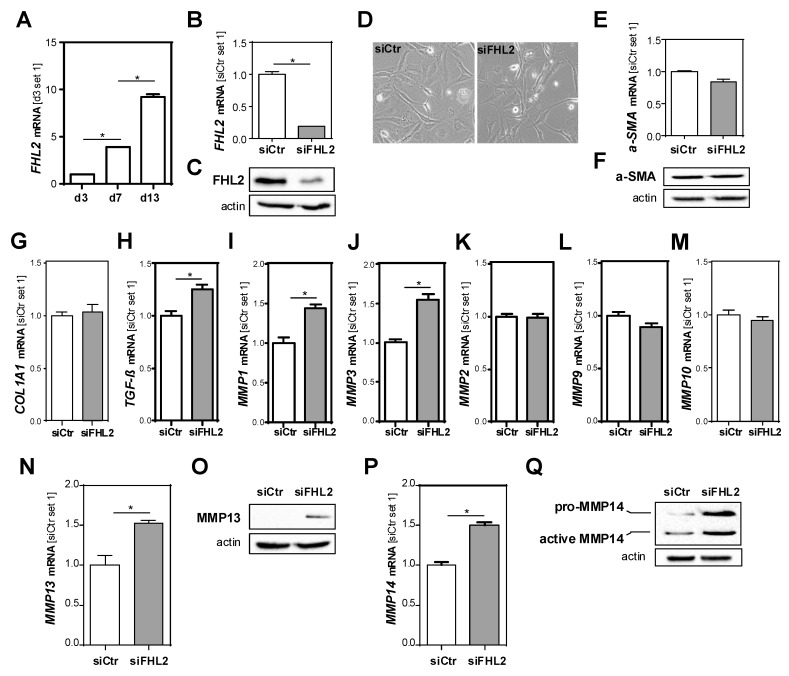
Effect of FHL2 depletion on pro-fibrogenic gene expression in hepatic stellate cells in vitro. (**A**) *FHL2* mRNA expression during activation of primary human hepatic stellate cells (HSCs). (**B**,**C**) FHL2 mRNA and protein expression in LX-2 cells transfected with si-pools against FHL2 (siFHL2) and si-control-pools (siCtr). (**D**) Representative microscopical images 72 h after transfection (10× magnification). (**E**,**F**) *α-SMA* mRNA and protein expression. (**G**) *COL1A1*, (**H**) *TGF-β*, (**I**) *MMP1*, (**J**) *MMP3*, (**K**) *MMP2*, (**L**) *MMP9* and (**M**) *MMP10* mRNA expression and (**N**,**O**) *MMP13* and (**P**,**Q**) *MMP14* mRNA and protein expression 72 h after transfection. (*: *p* < 0.05).

Besides hepatocellular injury and inflammation, enhanced fibrosis was the most prominent pathological feature of the Fhl2-ko mice in the BDL model compared to the wt mice. The activation of hepatic stellate cells (HSCs) is the key event of hepatic fibrosis [2,3]. Here, we found that *FHL2* expression was increased in primary human HSCs during in vitro activation (Figure 5A). 

To further assess whether the pro-fibrogenic effects of FHL2 deficiency in the BDL model were also mediated via direct effects in HSCs, we depleted FHL2 in the human HSC line LX-2 by applying si-pool technology. Control cells were transfected with unspecific control si-pools (Figure 5B,C). A microscopical analysis revealed no differences between the FHL2-depleted and control HSCs (Figure 5D); additionally, *α-SMA* mRNA and protein levels (Figure 5E,F), as well as *COL1A1* mRNA expression, were not affected by FHL2 depletion (Figure 5G).

In contrast, *TGF-β* levels were slightly but significantly higher in the FHL2-depleted cells compared to the control cells (Figure 5H). Furthermore, the mRNA expression of several metalloproteinases, including *MMP1, MMP3, MMP13* and *MMP14*, was higher in FHL2-depleted cells (Figure 5I–N,P). A western blot analysis of MMP13 and MMP14 revealed that these differences were even more pronounced on the protein level (Figure 5O,Q). In summary, these data indicate that FHL2 depletion also has direct pro-fibrogenic effects in HSCs in vitro. 

## 4. Discussion

In this study, we aimed to analyze the function of FHL2 in cholestatic liver injury. In murine studies, we used the BDL model, a well-established model to mimic cholestatic liver injury and fibrosis. In this model, the Fhl2-deficient mice revealed a significantly enhanced manifestation of pathological progression. A histological analysis and serum transaminase levels showed significantly enhanced hepatocellular damage. In a previous study, liver histology and serum transaminase levels did not differ between the Fhl2-deficient and wt mice in the model of chronic toxic liver injury with carbon tetrachloride (CCl_4_) [19]. This indicates that Fhl2 deficiency does not uniformly enhance the vulnerability of hepatocytes for (toxic) injury. Still, it has to be noted that here, as well as in the previous study that applied the CCl_4_ model, only male mice have been assessed. A study by Govoni et al. found that the female Fhl2-ko mice revealed a lower bone mineral content and bone mineral density compared to their male littermates [27]. Further studies need to evaluate whether there are also gender-specific differences in regard to Fhl2 and (cholestatic) liver injury.

Despite the effect on bone density, the Fhl2-ko mice have shown no obvious abnormalities, suggesting a high capacity for the fine-tuning adjustment and functional redundancy of Fhl2 under physiological conditions [9]. Additionally, here and as already described in previous studies [19], the Fhl2-ko control or sham-operated mice did not show any hepatologic abnormalities, and after BDL, a macroscopic analysis did not show pathological alterations besides the liver (data not shown). Still, it has to be considered that the complete Fhl2-ko in the mice may have also affected further organs or (patho)physiological mechanisms that contributed to the observed enhanced hepatocellular injury, inflammation and fibrosis in the BDL model.

Bile acids are significantly elevated after BDL and are majorly involved in cholestatic liver injury [28,29]. This prompted us to further assess the role of FHL2 in bile acid metabolism. We found that, under basal conditions, the FHL2-depleted hepatoma cells showed significantly higher *NTCP* expression levels compared to the control cells; additionally, upon bile acid stimulation, the expression of this major transporter for uptake of bile acids into hepatocytes remained significantly higher in the FHL2-depleted cells. Moreover, the FHL2-depleted cells showed a higher *CYP7A1* expression upon bile acid stimulation. CYP7A1 is the rate-limiting enzyme in bile acid synthesis. In combination, these findings indicate enhanced bile acid levels as potential reason for the more pronounced hepatocellular injury of the Fhl2-deficient mice in the BDL model.

To the best of our knowledge, no previous studies have assessed the role of FHL2 in bile acid metabolism. Only one study described the impact of FHL2 on cholesterol metabolism in vascular smooth muscle cells [30]. Kurakula et al. observed that cholesterol synthesis and liver X receptor (LXR) pathways are altered in the absence of FHL2, functionally resulting in an attenuated cholesterol efflux [30].

Next to enhanced hepatocellular injury, FHL2 deficiency caused a markedly enhanced expression of pro-inflammatory genes, paralleled by enhanced fibrogenesis in the BDL model. These pathological processes are closely intertwined, and it is difficult to dissect whether the enhanced fibrosis was indirectly caused by the more pronounced injury and inflammation or whether FHL2 also directly impacts hepatic fibrosis and HSCs.

To get further insight into this question, we compared HSCs with and without FHL2 depletion. In line with a previous study that showed an impact of FHL2 on *TGF-β* expression [31], we observed a slightly but significantly enhanced expression of this strong pro-fibrogenic cytokine in FHL2-depleted HSCs. Moreover, the expression of several MMPs was enhanced in the FHL2-depleted HSCs. These ECM-modulating enzymes are known to promote hepatic fibrogenesis as well as multiple aspects of liver inflammation [32,33]. Together, these data suggest that the lack of FHL2 in HSCs contributed to the enhanced fibrogenesis of the Fhl2-ko mice in the BDL model.

Moreover, it has to be considered that further parenchymal and non-parenchymal liver cells, as well as infiltrating immune cells, are involved in cholestatic liver injury [34]. Considering the pleiotropic role of FHL2, it appears likely that FHL2 also impacts cholestatic liver injury via its effect on these other cell types. For example, Dahan et al. found that the knockdown of FHL2 in macrophages impaired lipopolysaccharide-induced NF-κB activity [35]. Furthermore, FHL2 cannot be generally considered as beneficial. Rather, it has shown not only tissue- and cell context-specific functions but also often opposing functions. Thus, Nouët et al. described enhanced apoptosis and hepatocarcinogenesis upon Fhl2 overexpression in the liver of Fhl2 transgenic mice [36]. However, another study found delayed hepatocyte regeneration following partial hepatectomy in Fhl2-ko mice [35]. Our study has shown that a deficiency in FHL2 aggravates cholestatic liver injury, further underscoring the important role of FHL2 in liver homeostasis.

## Figures and Tables

**Table 1:** Primer sequences for quantitative real-time PCR.

Gene	Forward (5′-3′)	Reverse (5′-3′)
*18S*	TCTGTGATGCCCTTAGATGTCC	CCATCCAATCGGTAGTAGCG
**Human**		
*α-SMA*	CGTGGCTATTCCTTCGTTAC	TGCCAGCAGACTCCATCC
*BAX*	GGCCCACCAGCTCTGAGCAGA	GCCACGTGGGCGTCCCAAAGT
*BCL2*	GCGGATTTGAATCTCTTTCTC	CACTAAACTGACTCCAGCTG
*BSEP*	TGCCCAGAATGGCCCTACA	CCAGCATTGCCCTGAAACCA
*COL1A1*	CGGCTCCTGCTCCTCTT	GGGGCAGTTCTTGGTCTC
*CYP7A1*	CCATAAGGTGTTGTGCCACGG	TCCGTGAGGGAATTCAAGGCA
*FHL2*	GAAACTCACTGGTGGACAAGC	GTGGCAGATGAAGCAGGTCT
*MMP1*	TCACCAAGGTCTCTGAGGGTCAAGC	GGATGCCATCAATGTCATCCTGAGC
*MMP2*	GCTGGGAGCATGGCGATGGATACC	GGACAGAAGCCGTACTTGCCATCC
*MMP3*	TGCTGTTTTTGAAGAATTTGGGTT	CAATTCACAGAGACTTAGGTGAAGA
*MMP9*	TGCCTTTGGACACGCACG	CCTGGTTCAACTCACTCCGGG
*MMP10*	GGGGGAAGACAGATATGGGT	CTGTTCAGTGCAATTCAAAAGC
*MMP13*	TACCAGACTTCACGATGGCATTGCTG	AAAGTGGCTTTTGCCGGTGTAGGTG
*MMP14*	GGAACCCTGTAGCTTTGTGTCTGTC	TCTCTACCCTCAACAAGATTAGATTCC
*MRP2*	TCACATGTCCATCCACTGTTTCA	TGCTCAAAACAAGTGGCAGG
**Mouse**		
*α-sma*	CCAGCCATCTTTCATTGGGAT	CCCCTGACAGGACGTTGTTA
*Col1a1*	CTGTTCCAGGCAATCCACGA	ATCAGCTGGAGTTTCCGTGC
*Fhl2*	ACTGCCTGACCTGCTTCTGT	TTGCCTGGTTATGAAAGAAAA
*Hmox-1*	CACGCATATACCCGCTACCT	CCAGAGTGTTCATTCGAGCA
*Il-1*	TGCCACCTTTTGACAGTGATG	AAGGTCCACGGGAAAGACAC
*Mcp-1*	TGCAGGTCCCTGTCATGCTTC	TGGACCCATTCCTTCTTGGGG
*Mmp1*	CTTGGCCACTCCCTAGGTCT	AGGGCTGGGTCACACTTCTC
*Mmp2*	ATGGACAGCCCTGCAAGTTC	CAGTGGACATAGCGGTCTCG
*Mmp9*	GTCCAGACCAAGGGTACAGC	CTGTCGGCTGTGGTTCAGTT
*Pai-1*	ATGGGGCCGTGGAACAAGAA	AGGCGTGTCAGCTCGTCTAC
*Tgf-β*	CATTGCTGTCCCGTGCAGAG	CAGGCGTATCAGTGGGGGTC
*Tnf*	CCCTCACACTCAGATCATCTTCT	GCTACGACGTGGGCTACAG
